# Supplementary material for: Mobile Spatiotemporal Gait Segmentation Using an Ear-Worn Motion Sensor and Deep Learning
Source: Sensors (Basel). 2024 Oct 4;24(19):6442. doi: 10.3390/s24196442 (PMC11479375; doi:10.3390/s24196442)
Supplement: Supplementary file 1 [file sensors-24-06442-s001.zip › sensors-3192534-supplementary.pdf]

Supplementary Materials

Table S1. Detailed architecture of the temporal mEar model

layer	details
**input**	input dimension = (n, 3) (sample length, acceleration x,y,z [m/s^2])
**TCN blocks**	each block (number of blocks = 3) consists of:<br>• conv1d layer: kernel size = 5, dilation = exponentially increasing (2^i for block i), padding = automatically calculated for input/output length matching<br>• batch normalization: 1d normalization on 16 out channels<br>• ReLU activation<br>• dropout = 0.2
**final classifier**	conv1d layer:<br>• input channels = 16<br>• output channels = 2<br>• kernel size = 1
**activation**	sigmoid activation applied to the output of the classifier
**output**	output dimension = 2 per sequence<br>the model processes the input sequentially with temporal convolution, providing the same number of output steps as input, with a final binary classification per time step

Table S2. Detailed architecture of the two spatial mEar models

layer	details
**input**	input dimension = (n, 3) (sample length, acceleration x,y,z [m/s^2])
**TCN blocks**	each block (number of blocks = 3) consists of:<br>• conv1d layer: kernel size = 2, dilation = exponentially increasing (2^i for block i), padding = automatically calculated for input/output length matching<br>• chomp1d: removes excess padding from output<br>• ReLU activation<br>• dropout = 0.2
**residual connection**	skip connection between input and output of each block<br>if the input/output channels differ, downsampling is applied using a Conv1d layer.
**global average pooling**	adaptive average pooling over the temporal dimension, reducing the sequence length to 1
**fully connected layer**	linear layer that maps the final pooled output to the specified output size
**output**	output dimension = 1 (stride width / stride length)

Figure S1. Detailed graphs of (**A**) the temporal mEar model (example Sequence length = 320) and (**B**) the spatial mEar models (example Sequence length = 110)

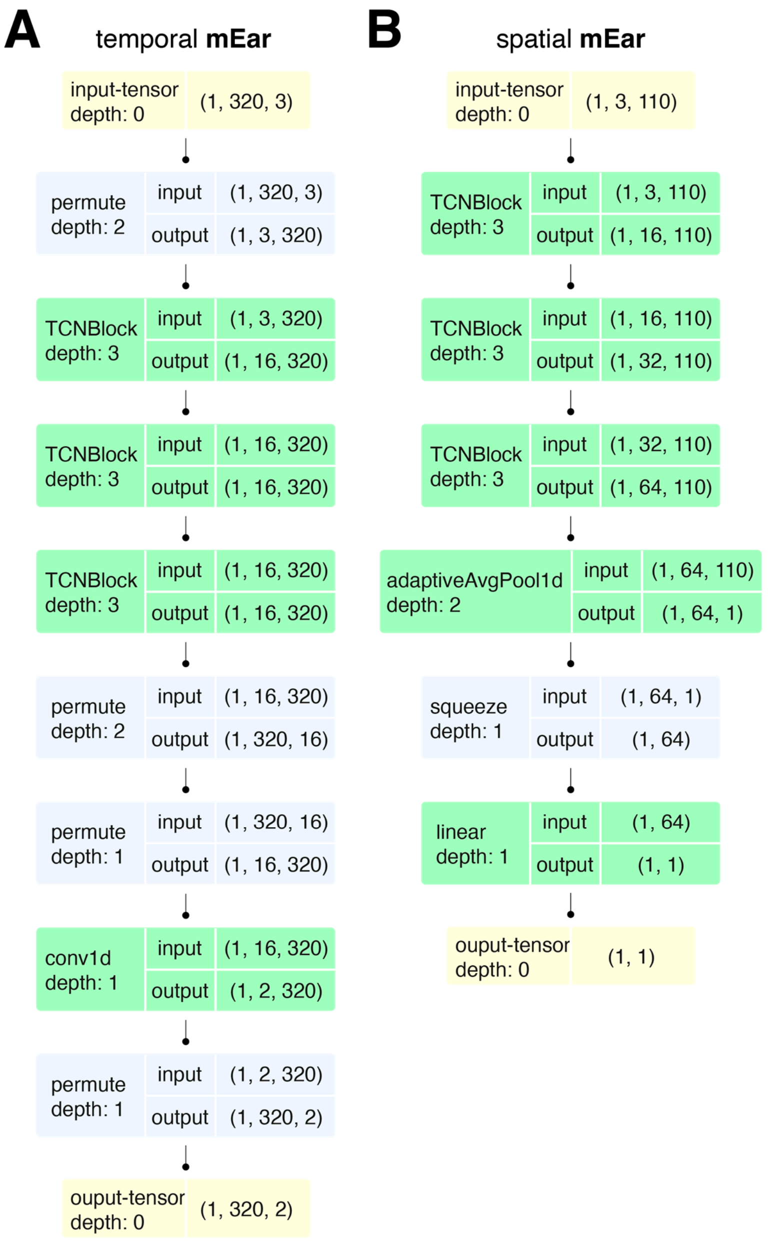

Table S3. Hyperparameter grid search

model	temporal mEar	spatial mEar
**batch size options**	[1, 4, 8, 12]	[1, 4, 8, 12]
**learning rate options**	[1e-3, 1e-4, 1e-5]	[1e-3, 1e-4, 1e-5]
**epoch options**	[15, 30, 75]	[10, 30, 75]
**best results**	batch size: 1<br>learning rate: 1e-03<br>epochs: 15	batch size: 12<br>learning rate: 0.001<br>epochs: 30
